# Establishment of a Loop-Mediated Isothermal Amplification (LAMP) Method for the Detection of *Fusarium oxysporum* f. sp. *momordicae*

**DOI:** 10.3390/jof12050378

**Published:** 2026-05-20

**Authors:** Xiongjuan Huang, Chengcheng Feng, Xixi Ju, Yuhui Huang, Xiaofeng Chen, Jiazuo Liang, Xinglian Liu, Zhendong Chen, Rukui Huang

**Affiliations:** 1 Vegetable Research Institute, Guangxi Academy of Agricultural Sciences, Nanning 530007, China; hxj1102@gxaas.net (X.H.); fengchengcheng@gxaas.net (C.F.); jxx@gxaas.net (X.J.); yuhui@gxaas.net (Y.H.); cxf@gxaas.net (X.C.); jiazuoliang@gxaas.net (J.L.); 1887715572@139.com (X.L.); 2 Guangxi Academy of Agricultural Sciences, Nanning 530007, China

**Keywords:** *Fusarium oxysporum* f. sp. *momordicae*, loop-mediated isothermal amplification (LAMP), visual detection, specific detection, bitter gourd, Fusarium wilt

## Abstract

Bitter gourd (*Momordica charantia* L.) is an important vegetable and medicinal crop in tropical/subtropical regions, but suffers severe yield losses (even total failure) from Fusarium wilt caused by *Fusarium oxysporum* f. sp. *momordicae* (*Fom*). There is no specific detection system available to detect this pathogen, and the methods used for other pathogens exhibit cross-reactivity and require specialized equipment. Therefore, this study developed a loop-mediated isothermal amplification (LAMP) assay for early *Fom* diagnosis. Initially, five sets of LAMP primers targeting the conserved regions of *Fom*, located within the region amplified by the FOMM-SPF/SPR PCR primers, were tested for specificity and sensitivity. In this experiment, FoM-1-2 showed optimal specificity, identifying 44 *Fom* strains without cross-reactivity with 10 other non-*Fom* species after a 60 min incubation at 64 °C. A visual readout based on a fluorescent dye (green for positive, pale orange for negative) eliminated the need for gel electrophoresis and specialized instruments. The LAMP assay was 100-fold more sensitive than conventional PCR (detection limit: 5.6 pg/μL vs. 560 pg/μL). In inoculated seedlings, LAMP detected *Fom* in basal stems at four days post-inoculation and top leaves at six days, whereas conventional PCR yielded faint bands in the basal stem after eight days. Moreover, LAMP enabled non-destructive detection. Thus, the present study developed a rapid, specific, and sensitive visual LAMP assay, supporting early diagnosis of bitter gourd Fusarium wilt.

## 1. Introduction

Bitter gourd (*Momordica charantia* L.) is a herbaceous vine of the Cucurbitaceae family. It originated in Africa and was domesticated in Asia before spreading widely to the tropical, subtropical, and temperate regions [1]. Currently, it is valued and cultivated worldwide as a nutritious vegetable rich in vitamins, polyphenols, and bioactive compounds [2]. It has also been used in traditional medicine due to its blood-glucose-lowering and antioxidant activities [3]. Asia accounts for over 70% of global production, with India being the largest producer, followed by large-scale cultivation in southern China (Guangxi, Guangdong, Fujian) and commercial production in several other Asian countries [4,5].

In recent years, the global cultivation area has increased, but continuous cropping has led to agronomic challenges such as soil degradation, intensified soil-borne diseases, and reduced yield potential across major production regions. Among the soil-borne pathogens, *Fom*, the causal agent of bitter gourd Fusarium wilt, is a destructive one, infecting the plant’s vascular system and disrupting nutrient and water transport. Fusarium wilt, which causes wilting and plant death, is a major challenge that directly leads to yield loss. In greenhouse continuous cropping areas of Guangxi, an incidence rate of about 40.35–80.84% has been reported during the full fruiting stage, leading to complete crop failure in extreme cases [6]. Similarly, in the major bitter gourd-producing regions of India and Southeast Asia, the yield reduction rate due to the disease generally exceeds 30% [7].

Typically, the pathogen *Fom* spreads through soil, where it can survive for 6–7 years. Continuous cropping of bitter gourd promotes the accumulation of this pathogen in the soil, further exacerbating the damage [8]. The fruit from infected plants exhibits poor development and malformed appearance, leading to a significant reduction in commercial value. Metabolomic studies have shown that Fusarium wilt in bitter gourd disrupts phenylpropanoid biosynthesis, thereby reducing secondary metabolite content [6]. This reduction impairs both the medicinal value and market competitiveness of the produce. Due to the scarcity of resistant cultivars, Fusarium wilt has emerged as a dominant soil-borne disease of bitter gourd [9,10]. Therefore, establishing a highly efficient and rapid method to detect *Fom*.

Recent advances in comparative genomics have highlighted the Secreted in Xylem (*SIX*) gene family as key determinants of host specificity in *Fusarium oxysporum* [11]. These effector genes exhibit pronounced sequence variation and distinct presence/absence patterns among different formae speciales, making them ideal targets for developing forma specialis-specific molecular diagnostics [12,13,14]. This knowledge provides a rational basis for targeting genomic regions with low inter-forma speciales homology to achieve specific detection.

Loop-mediated isothermal amplification (LAMP) is a nucleic acid amplification technique used to detect various pathogens, such as bacteria, fungi, and viruses [15]. It uses the *Bacillus stearothermophilus* (Bst) DNA polymerase to synthesize and amplify the pathogen’s DNA by strand displacement under isothermal conditions (60–65 °C). The method employs 4–6 primers to recognize six specific regions in the pathogen’s DNA and initiates exponential amplification via self-priming stem-loop structures [15]. The outer primers F3/B3 target the outer specific regions of the target DNA, inner primers FIP/BIP target the inner specific regions, and optional loop primers target the amplicon’s loop structures. Research has proven that LAMP rapidly amplifies the target DNA with high specificity and efficiency. It operates under isothermal conditions without the need for expensive thermal cyclers, offers rapid amplification (typically 30–60 min), and exhibits high specificity via multi-primer targeting of unique DNA regions. Compared with conventional PCR-based methods such as SCAR markers and URP-PCR, LAMP offers the advantages of isothermal amplification, visual readout, and field applicability, addressing the limitations of laboratory dependence and potential cross-reactivity. Therefore, it is currently used to detect various plant pathogens, such as *Fusarium oxysporum* f. sp. *niveum* (*FON*) [16], *Fusarium oxysporum* f. sp. *cucumerinum* Owen (*FOC*) [17], *Fusarium oxysporum* f. sp. *cubense* (*FOC*) [18], and various other wilt-causing *Fusarium oxysporum* strains. However, LAMP has not been used to detect *Fom*.

Therefore, the present study aimed to develop a rapid LAMP-based visual detection system for *Fom* by targeting a genomic region with low homology to other formae speciales. The performance of this method, including its specificity against closely related *F. oxysporum* formae speciales, sensitivity for early infection, accuracy in field detection, and applicability under real production conditions, was evaluated in parallel with conventional PCR using the *Fom*-specific primers FOMM-SPF/SPR. The study’s findings will facilitate targeted management of *Fom* and help mitigate field losses due to Fusarium wilt in commercial bitter gourd plantations.

## 2. Methods

### 2.1. Test Strains

A total of 44 strains of *Fom* were used in this study. These strains were isolated, identified, and preserved by the Vegetable Research Institute of Guangxi Academy of Agricultural Sciences (VRI, GXAAS). Additionally, 10 strains of other pathogens, including *Fusarium oxysporum* f. sp. *melonis* and *Fusarium oxysporum* f. sp. *cucumerinum*, were obtained from the China General Microbiological Culture Collection Center (CGMCC), the Agricultural Culture Collection of China (ACCC), or the Vegetable Research Institute of Guangxi Academy of Agricultural Sciences (VRI, GXAAS) for the study. Details of these strains are shown in Table 1.

### 2.2. Fungal Culturing and Nucleic Acid Extraction

The fungal strains were inoculated on potato dextrose agar (PDA) medium and cultured at 30 °C for 7 days. The mycelia that grew on PDA were collected and used to extract total DNA with the Biospin Fungal Genomic DNA Extraction Kit (Hangzhou Bioer Technology Co., Ltd., Hangzhou, China). The quality and concentration of the extracted DNA were determined using a UV-visible spectrophotometer (GENESYS 10S, Thermo Fisher Scientific, Waltham, MA, USA). Absorbance was measured at 260 nm and 280 nm. DNA purity was assessed by the A260/A280 ratio, with values of 1.8–2.0 considered as pure. DNA concentration (ng/μL) was calculated using the formula: A260 × 50× dilution factor. Finally, the samples were stored at −20 °C for subsequent use.

### 2.3. Primer Designing and Synthesis

LAMP primers, for the specific detection of *Fom*, were designed based on the conserved regions targeted by the PCR primers FOMM-SPF/SPR (Table 2). The approach ensured that the regions targeted in LAMP were encompassed within those amplified by the PCR primers of *Fom*. Importantly, preliminary sequence analysis confirmed that these target regions exhibited low homology to corresponding sequences in other *Fusarium oxysporum* formae speciales and related fungal pathogens, thereby providing a basis for achieving specific detection. Five sets of LAMP primers (FoM-1-1, FoM-1-2, FoM-3-1, FoM-3-2, and FoM-3-3) were designed using the online LAMP primer design software Primer Explorer (http://primerexplorer.jp/e/index.html, accessed on 18 March 2020). Among these, FoM-1-1 included a pair of inner primers, a pair of outer primers, and a backward loop primers; FoM-1-2 included a pair of inner primers and a pair of outer primers; FoM-3-1 included a pair of inner primers, a pair of outer primers, and a pair of loop primers; FoM-3-2 included a pair of inner primers, a pair of outer primers, and a forward loop primer; FoM-3-3 included a pair of inner primers and a pair of outer primers (Table 2). These primers were synthesized at Sangon Biotech Co., Ltd. (Shanghai, China), dissolved in ddH_2_O, and stored at −20 °C for later use.

### 2.4. In Silico Validation of the Fom-Specific Marker

To verify the novelty and specificity of the marker sequence obtained in this study, BLAST searches were performed against the NCBI GenBank database using the online BLAST tool (https://blast.ncbi.nlm.nih.gov, accessed on 18 May 2025) (E-value ≤ 1 × 10^−5^, coverage ≥80% considered a valid match). Additionally, local BLASTn alignments were conducted using BLAST+ (v2.14.0) against the genome sequences of standard strains of *F. oxysporum* f. sp. *niveum* (*FON*), f. sp. *melonis* (*Fomel*), f. sp. *cucumerinum* (*FOCu*), and f. sp. *cubense TR4* (*FOC*) obtained from the Agricultural Culture Collection of China. The complete sequence of the marker is shown in Table 3.

### 2.5. LAMP Reaction

The LAMP reaction system (25 μL) included 12.5 μL of 2× reaction buffer, 1 μL of Bst DNA polymerase, 1 μL each of forward inner primer (FIP, 1.6 μmol/L), backward inner primer (BIP, 1.6 μmol/L), forward outer primer (F3, 0.8 μmol/L), backward outer primer (B3, 0.8 μmol/L), loop forward primer (LF, 0.2 μmol/L), and loop backward primer (LB, 0.2 μmol/L), 1 μL of the fluorescent visual detection reagent, 2 μL of the template DNA, and water. All the above reagents were purchased from Toyobo Biotech Co., Ltd. (Shanghai, China). The reaction mixture was incubated at a constant temperature of 64 °C for 60 min, and turbidity was measured using an LA-320C Isothermal Amplification Real-Time Turbidity Detector (Eiken Chemical Co., Ltd., Tokyo, Japan) every 6 s from 0 to 60 min. The optimal primer set was screened based on the real-time amplification curve. In the assay, genomic DNA from *Fom* was used as the positive control, and deionized water was used as the negative control.

### 2.6. Analysis of LAMP Specificity

A total of 44 strains of *Fom* and 10 strains of non-*Fom* (Table 1) were used to test the specificity of the LAMP-based detection assay. LAMP was carried out in a reaction system, as explained in the previous section, using the genomic DNA of *Fom* as the positive control and deionized water as the negative control. The turbidity of the reaction mixture was measured over time using the real-time turbidimeter, and the amplification curves for these samples were plotted. Additionally, fluorescence visualization was also carried out; green indicated the presence of the pathogen (a positive result), while pale orange indicated the absence of the pathogen (a negative result).

Conventional PCR-based amplification was performed using DNA from a subset of the total 44 strains of *Fom* and 10 strains of non-*Fom* (Table 1), with the FOMM-SPF and FOMM-SPR primers (Table 2) at the same concentration as in the LAMP assay. The reaction was carried out on a Gradient PCR Instrument (Analytik Jena AG, Jena, Germany) in a 25 μL mixture containing 12.5 μL of 2× Green Taq Master Mix, 1 μL each of FOMM-SPF and FOMM-SPR primers (2.5 μmol/L), 1 μL of template DNA, and ddH_2_O to top up the volume. The PCR program was set as follows: initial denaturation at 94 °C for 3 min, followed by 30 cycles of denaturation at 94 °C for 15 s, annealing at 57 °C for 30 s, extension at 72 °C for 20 s, and a final extension at 72 °C for 5 min. The amplicons were detected by electrophoresis on a 1.5% agarose gel, and the presence of a 294 bp band was considered a positive result. The PCR results were finally compared with the fluorescence-based visual results of LAMP. Further, both PCR and LAMP were tested on 44 strains of *Fom* (target strains) and 10 strains of non-*Fom* (non-target strains, Table 1), to assess their specificity; here, positive amplification for target strains and no amplification for non-target strains indicated good specificity.

### 2.7. Analysis of LAMP Sensitivity

The genomic DNA of the FOM4501 strain was serially diluted (56 ng/μL, 5.6 ng/μL, 560 pg/μL, 56 pg/μL, 5.6 pg/μL, 560 fg/μL, and 56 fg/μL) and used to determine the sensitivity of the LAMP method. Three replicates were set for each concentration, and 1 μL of template was used in each reaction. Here, deionized water served as the negative control. Then, the turbidity of the reaction mixture was measured over time using a real-time turbidimeter, and amplification curves were plotted to analyze amplification kinetics, compare positive reaction signals across different template concentrations, and ultimately confirm the limit of detection (LOD) of the assay. The visual detection was carried out using the fluorescent dye calcein.

PCR-based amplification was carried out using the FOMM-SPF and FOMM-SPR primers (Table 2) for DNA at the same concentration as in the LAMP sensitivity assay. The method was carried out in the same manner as described in the LAMP specificity section. The PCR results were subsequently compared with the LAMP visual results to assess sensitivity.

### 2.8. Detection of Fom in Different Parts of Infected Bitter Gourd Seedlings by LAMP

The FOM4501 strain was used to prepare a *Fom* spore suspension at a concentration of 1 × 10^6^ CFU/mL. A highly susceptible bitter gourd inbred line, MC10-1 (provided by the Vegetable Research Institute, Guangxi Academy of Agricultural Sciences), was used. Bitter gourd seedlings at the four-leaf-one-heart stage were uprooted from seedling trays, shaken to remove the root substrates, rinsed with sterile water, and then set aside. These seedlings were inoculated with the pathogen by irrigating the roots with the *Fom* spore suspension for approximately 30 min. After inoculation, the seedlings were replanted into the seedling trays.

At 0, 2, 4, 6, 8, 10, 12, and 15 days after inoculation, the first and second true leaves from the base of the seedlings, the first and second expanded leaves from the top, and the stem 0.5 cm above the soil surface were collected. After washing with clean water, each plant part was used to extract DNA with the Biospin Fungal Genomic DNA Extraction Kit (Hangzhou Bioer Technology Co., Ltd., Hangzhou, China).

Then, LAMP and conventional PCR were performed, using the DNA of the FOM4501 strain as the positive control, DNA extracted from healthy (non-inoculated) bitter gourd seedlings, and deionized water as the negative control. Both assays were carried out as described in the earlier sections. Then, the fluorescence visual detection results and the PCR results were compared. The limit of detection (LOD) values of the LAMP and PCR methods were compared, and the consistency of positive/negative results across all concentration gradients was evaluated to verify the applicability of the detection system.

## 3. Results

### 3.1. In Silico Validation of Marker Specificity

The complete sequence of the *Fom*-specific marker obtained in this study (293 bp) is shown in Table 3. BLAST search against the NCBI GenBank database revealed no significant homologous matches under stringent criteria (E-value ≤ 1 × 10^−5^, coverage ≥80%), confirming that this sequence has not been previously reported and exhibits good novelty.

Local alignments against genomes of closely related *Fusarium oxysporum* f. sp. *momordicae* (*Fom*) demonstrated substantial sequence divergence (Table 3). The consistently low coverage (<45%) with multiple gaps, and the absence of a valid match in one forma specialis (similarity <60%), indicate that this marker resides in a region of pronounced genomic variability. This divergence is particularly noteworthy because it occurs in a region expected to be conserved among formae speciales—suggesting that the marker may be located within a lineage-specific genomic island associated with host adaptation. Such regions have been shown to exhibit accelerated evolution and structural variation, providing ideal targets for forma specialis-specific diagnostics [11,12,13,14]. The marked specificity observed at the sequence level provides a strong foundation for the subsequent development of a highly specific LAMP assay.

### 3.2. Screening of LAMP Primers

In this study, five primer sets (FoM-1-1, FoM-1-2, FoM-3-1, FoM-3-2, and FoM-3-3; Table 2) were tested for effectiveness in loop-mediated isothermal amplification (LAMP), and the optimal primer combination was selected based on turbidity values.

### 3.3. Verification of FoM-1-1 and FoM-1-2 Primers

**Figure 1:**
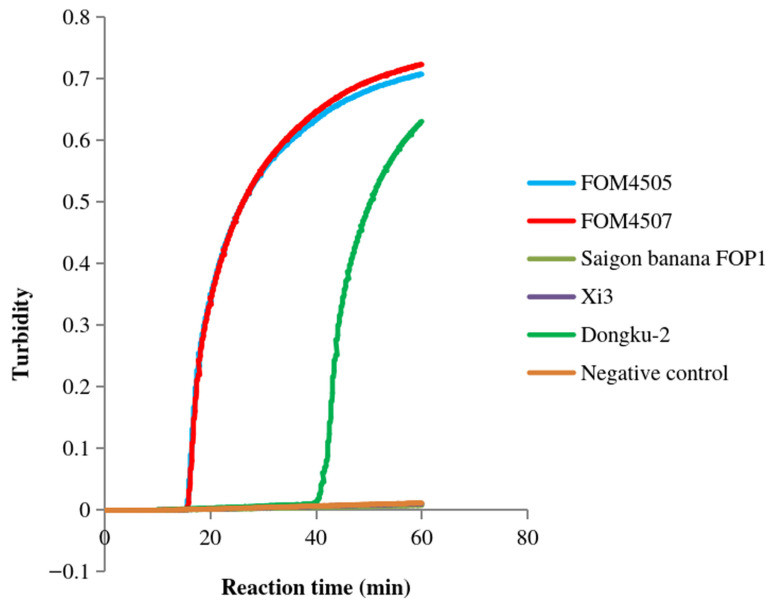
Specificity of FoM-1-1 primers in LAMP. The amplification curves of the *Fom* samples FOM4505 and FOM4507, the non-*Fom* samples Saigon banana FOP1, Xi3, and Dongku-2, and the negative control (deionized water) are shown in the figure.

**Figure 2:**
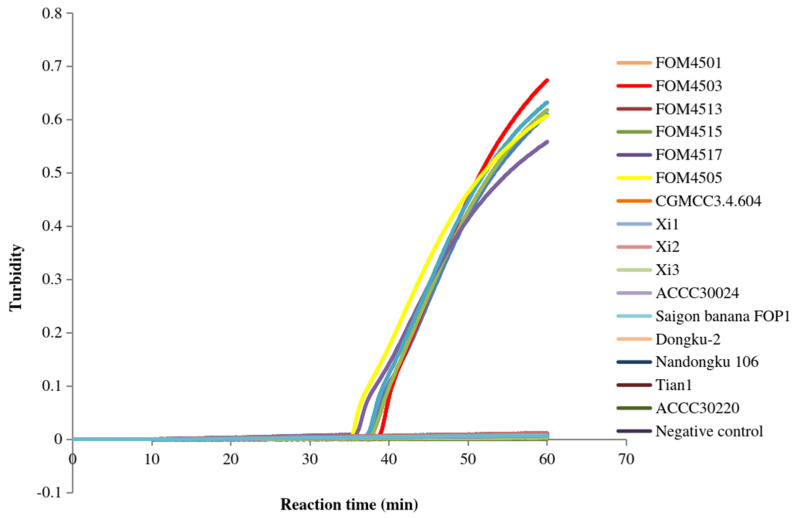
Specificity of FoM-1-2 primers in LAMP. The amplification curves of the *Fom* samples FOM4501, FOM4503, FOM4513, FOM4515, FOM4517, and FOM4505, the non-*Fom* samples CGMCC3.4.604, Xi1, Xi2, Xi3, ACCC30024, Saigon banana FOP1, Dongku-2, Nandongku 106, Tian1, and ACCC30220, and the negative control (deionized water) are shown.

We first analyzed the following two LAMP primer sets: primer FoM-1-1, containing a pair of inner primers, a pair of outer primers, and a backward loop primer; and primer FoM-1-2, including the same pair of inner primers and a pair of outer primers as FoM-1-1. After incubation at 64 °C for 60 min, the FoM-1-1 primer set detected the positive *Fom* samples (FOM4505 and FOM4507) but not the negative control (deionized water) and the other non-*Fom* samples (Figure 1). However, this primer set cross-reacted with Dongku-2, a strain belonging to a non-Momordicae specialized form of *F. oxysporum*. Similarly, the FoM-1-2 primer set detected the positive *Fom* samples (FOM4501, FOM4503, FOM4513, FOM4515, FOM4517, and FOM4505) (Figure 2). Unlike FoM-1-1, FoM-1-2 showed no cross-reactivity with any tested non-*Fom* samples (CGMC3.4.604, Xi1, Xi2, Xi3, Saigon banana FOP1, ACCC30024, Dongku-2, Nandongku 106, Tian1, and ACCC30220) or the negative control (deionized water). These results indicated that FoM-1-2 possessed excellent specificity for *Fom*, whereas FoM-1-1 lacked sufficient specificity due to cross-reactivity. Thus, FoM-1-2 was selected as the primer set to detect *Fom* via LAMP in subsequent experiments.

### 3.4. Verification of FoM-3-1, FoM-3-2, and FoM-3-3 Primers

**Figure 3:**
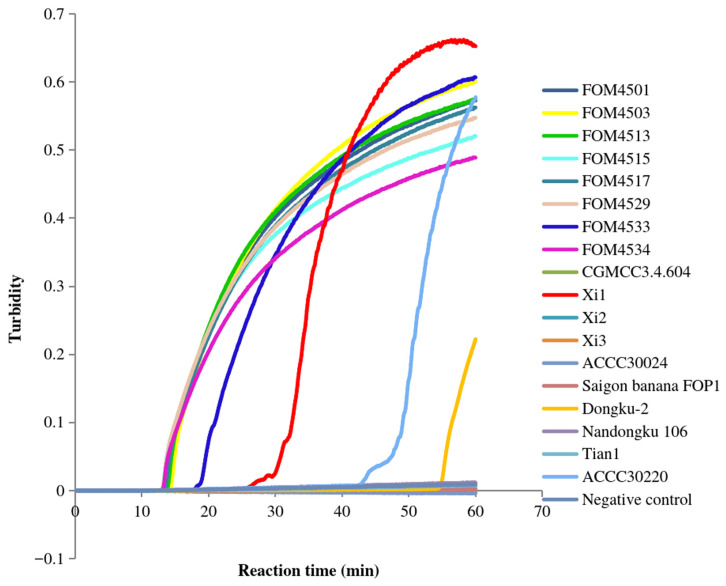
Specificity of FoM-3-1 primers in LAMP. The amplification curves of the *Fom* samples FOM4501, FOM4503, FOM4513, FOM4515, FOM4517, FOM4529, FOM4533, and FOM4534, the non-*Fom* samples CGMCC3.4.604, Xi1, Xi2, Xi3, ACCC30024, Saigon banana FOP1, Dongku-2, Nandongku 106, Tian1, and ACCC30220, and the negative control (deionized water) are shown.

**Figure 4:**
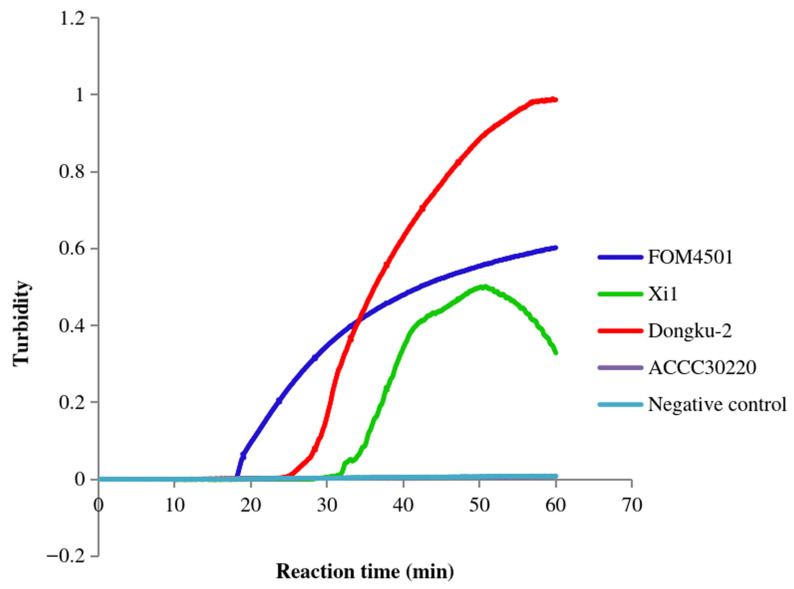
Specificity of FoM-3-2 primers in LAMP. The amplification curves of the *Fom* samples FOM4501, the non-*Fom* samples Xi1, Dongku-2, and ACCC30220, and the negative control (deionized water) are shown.

**Figure 5:**
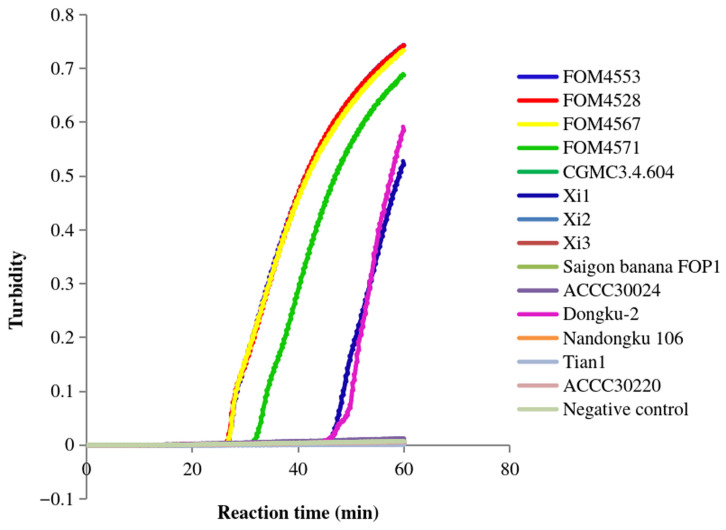
Specificity of FoM-3-3 primers in LAMP. The amplification curves of the *Fom* samples FOM4553, FOM4528, FOM4567 and FOM4571, the non-*Fom* samples CGMCC3.4.604, Xi1, Xi2, Xi3, Saigon banana FOP1, ACCC30024, Dongku-2, Nandongku 106, Tian1, and ACCC30220, and the negative control (deionized water) are shown

Further, we verified the FoM-3-1 primer set, containing a pair of inner primers, a pair of outer primers, and a pair of loop primers, the FoM-3-2 primer set, containing a pair of inner primers, a pair of outer primers, and a forward loop primer, and the FoM-3-3 primer set, containing a pair of inner primers and a pair of outer primers (Table 2). After incubation at 64 °C for 60 min, the FoM-3-1 primers detected the positive *Fom* samples (FOM4501, FOM4503, FOM4513, FOM4515, FOM4517, FOM4529, FOM4533, and FOM4534) but not the negative control (deionized water) and other non-*Fom* samples except for Xi1, Dongku-2, and ACCC30220 (Figure 3). After a similar isothermal incubation (60 min at 64 °C), the FoM-3-2 primers detected the positive samples (FOM4501) (Figure 4). These primers exhibited signals with Xi1 and Dongku-2 but not with the negative control. Meanwhile, the FoM-3-3 primers detected the positive *Fom* samples (FOM4553, FOM4528, FOM4567, and FOM4571) along with two non-*Fom* samples (Xi1 and Dongku-2) but not the negative control (deionized water) (Figure 5). The primers did not produce a signal with the negative control or the other non-*Fom* samples (CGMCC3.4.604, Xi2, Xi3, ACCC30024, Saigon banana FOP1, Nandongku 106, Tian1, and ACCC30220). These observations suggested that the FoM-3-1, FoM-3-2, and FoM-3-3 primer sets lack specificity for *Fom*.

### 3.5. Specificity of LAMP

The FoM-1-2 primers exhibited excellent specificity in detecting *Fusarium oxysporum* via the LAMP reaction in this study. To ensure the accuracy and reliability of this method, we conducted conventional polymerase chain reaction (PCR) with primers FOMM-SPF/SPR, LAMP turbidity assay with primers FoM-1-2, and LAMP visualization assay with primers FoM-1-2 on the 44 *Fom* samples (Table 1), the 10 non-*Fom* samples (Table 1), the positive control (FOM4501/FOM4523), and the negative control (deionized water), and compared their performance in terms of consistency and specificity.

**Figure 6:**
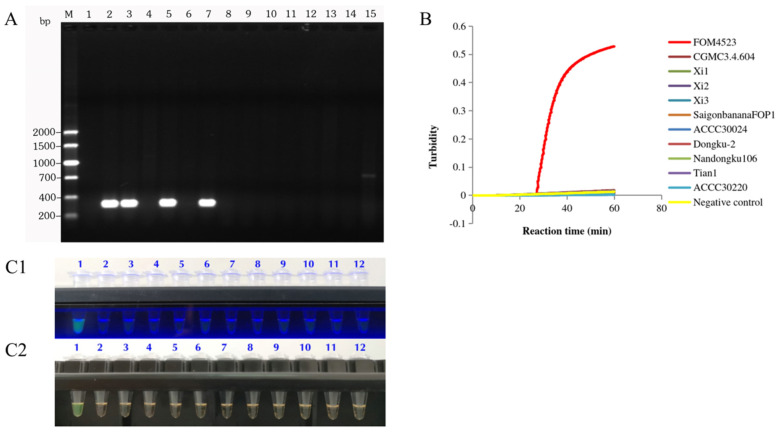
Specificity of conventional PCR (**A**), LAMP turbidity assay (**B**), and LAMP visualization assay ((**C1**), ultraviolet light; (**C2**), natural light) in detecting *Fom* with FoM-1-2 primers. Samples shown in (**A**) are as follows: M represents the DL2000 DNA Marker; 1. Deionized water (negative control); 2. FOM4501 (positive control); 3. FOM4503; 4. CGMCC3.4.604; 5. FOM4513; 6. Xi1; 7. FOM4515; 8. Xi2; 9. Xi3; 10. ACCC30024; 11. Saigon banana FOP1; 12. Dongku-2; 13. Nandongku 106; 14. Tian1; 15. ACCC30220. Samples shown in (**B**) are as follows: FOM4523. Non-*Fom* samples (CGMCC3.4.604, Xi1, Xi2, Xi3, ACCC30024, Saigon banana FOP1, Dongku-2, Nandongku 106, Tian1, and ACCC30220) and deionized water (negative control). (**C1**,**C2**) show the LAMP visualization results obtained with the fluorescent detection kit after a 60 min incubation at 64 °C. Here, green indicates a positive result, while pale orange indicates a negative result. The samples shown in (**C1**,**C2**) are as follows: 1. *Fom* (positive control, FOM4501); 2–5. *Fusarium oxysporum* f. sp. *niveum* (melon wilt pathogen); 6. *Fusarium oxysporum* f. sp. melonis; 7. *Fusarium oxysporum* f. sp. *cucumerinum*; 8 and 9. *Fusarium oxysporum* f. sp. *benincasae*; 10. *Fusarium oxysporum* f. sp. *cubense*; 11. *Fusarium solani* (non-pathogenic isolate); 12. deionized water (negative control).

After amplification via conventional PCR, the specific band (294 bp; agarose gel) was detected in the positive control (FOM4501) and other positive samples (FOM4503, FOM4513, and FOM4515) (Figure 6A), while no band was observed in the remaining samples or the negative control. Meanwhile, in the LAMP turbidity assay (Figure 6B) and the LAMP visualization assay (Figure 6(C1,C2)) after incubation at 64 °C for 60 min, the positive control (FOM4501) exhibited a green color under both UV light (Figure 6(C1)) and natural light (Figure 6(C2)). Meanwhile, the negative control and the 10 non-*Fom* samples exhibited a pale orange color (Figure 6(C1,C2)).

Further analysis revealed that the specificity curve from the LAMP turbidity assay (Figure 6B) aligned with the detection results of conventional PCR (Figure 6A) and LAMP visualization assay (Figure 6(C1,C2)). These observations indicate that the LAMP-based method (validated by conventional PCR) accurately and specifically detects *Fom*, and conventional PCR validated these results. Importantly, the in silico validation data presented in Table 3 are fully consistent with these experimental observations, further confirming that the selected marker originates from a highly specific region of the *Fom* genome.

### 3.6. Sensitivity of LAMP

To assess the sensitivity of the LAMP assay in detecting *Fom*, LAMP turbidity assay, LAMP visualization assay, and conventional PCR were carried out with the FoM-1-2 primers using the FOM4501 DNA at concentrations ranging from 56 ng/μL to 5.6 pg/μL. The same readout methods (LAMP turbidity, visualization, and conventional PCR) used in the specificity validation were employed here to ensure consistency.

**Figure 7:**
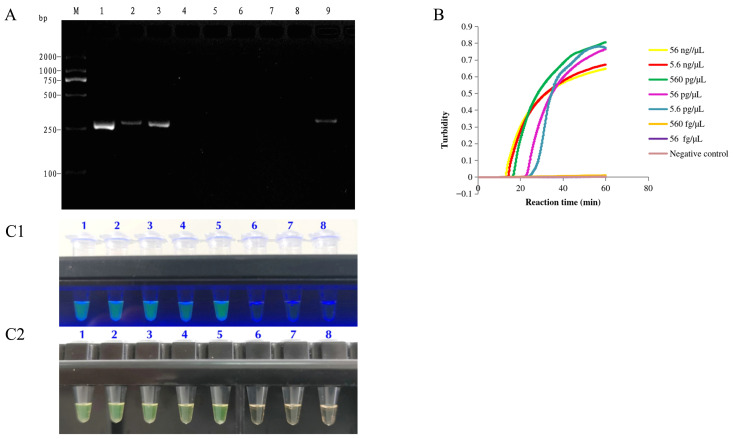
Sensitivity of conventional PCR (**A**), LAMP turbidity assay (**B**), and LAMP visualization assay ((**C1**), ultraviolet light; (**C2**), natural light) in detecting *Fom* using FoM-1-2 primers. The samples shown in (**A**) are as follows: M represents the DL2000 DNA Marker. 1~7: 56 ng/μL, 5.6 ng/μL, 560 pg/μL, 56 pg/μL, 5.6 pg/μL, 560 fg/μL, and 56 fg/μL of FOM4501 DNA; 8: deionized water (negative control); 9: 56 ng/μL of FOM4501 DNA (positive control). The samples shown in (**B**) are as follows: 1–7: 56 ng/μL, 5.6 ng/μL, 560 pg/μL, 56 pg/μL, 5.6 pg/μL, 560 fg/μL, and 56 fg/μL of FOM4501 DNA; 8: deionized water (negative control). (**C1**,**C2**) show the results after a 60 min incubation at 64 °C. Here, green indicates a positive result, while pale orange indicates a negative result. The samples shown in (**C1**,**C2**) are as follows: 1–7: 56 ng/μL, 5.6 ng/μL, 560 pg/μL, 56 pg/μL, 5.6 pg/μL, 560 fg/μL, and 56 fg/μL of FOM4501 DNA; 8: deionized water (negative control).

The amplification curves from the LAMP turbidity assay generated using the FOM4501 DNA at concentrations of 56 ng/μL, 5.6 ng/μL, 560 pg/μL, 56 pg/μL, and 5.6 pg/μL exhibited the typical sigmoidal shape (Figure 7B). Under both UV and natural light, FOM4501 DNA at 56 ng/μL, 5.6 ng/μL, 560 pg/μL, 56 pg/μL, and 5.6 pg/μL concentrations exhibited a green color, while that at 560 fg/μL and 56 fg/μL showed a pale orange color (Figure 7(C1,C2)). These observations indicated that the results based on the visual analysis are consistent with the LAMP turbidity curve (Figure 7B). Notably, the LAMP assay’s minimum detection limit was 5.6 pg/μL, which is 100-fold lower than that of conventional PCR (560 pg/μL).

Subsequently, the sensitivity of the LAMP assay evaluated using turbidity curves and visual inspection was compared with that of conventional PCR. We detected prominent target bands after conventional PCR with FOM4501 DNA at concentrations 56 ng/μL, 5.6 ng/μL, and 560 pg/μL, but not at concentrations less than or equal to 56 pg/μL (Figure 7A), indicating a minimum detection level of 560 pg/μL for conventional PCR. These observations thus proved that the sensitivity of the established LAMP assay was 100-fold higher than that of conventional PCR.

### 3.7. Detection of Fom in Different Parts of Infected Bitter Gourd Seedlings by the LAMP System

**Figure 8:**
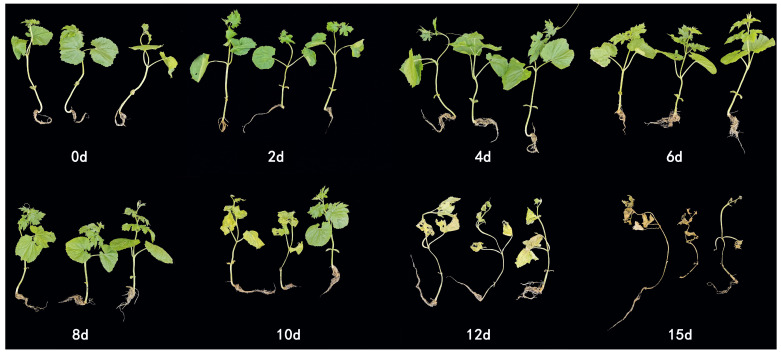
Symptoms of bitter gourd seedlings infected with *Fom*. The image shows the seedlings at 0, 2, 4, 6, 8, 10, 12, and 15 days after pathogen inoculation.

Finally, bitter gourd seedlings were infected with *Fom* and analyzed to test the applicability of the LAMP-based assay established in this study. Typical wilt symptoms, such as leaf yellowing and stem base rot, were observed during the infection period (Figure 8). Therefore, DNA was extracted from the seedling tissues at 0, 2, 4, 6, 8, 10, 12, and 15 days post-inoculation to detect the pathogen using the established LAMP method and the conventional PCR methods.

**Figure 9:**
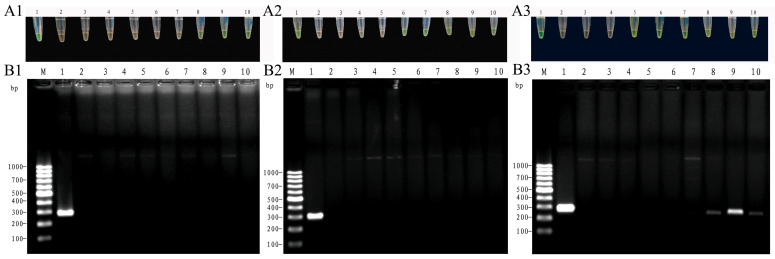
LAMP-based detection of *Fom* (*Fusarium oxysporum* f. sp. *momordicae*) in infected bitter gourd tissues using LAMP visualization assay ((**A1**): basal leaves; (**A2**): top leaves; (**A3**): stem base) and corresponding conventional polymerase chain reaction (PCR) with agarose gel electrophoresis ((**B1**): basal leaves; (**B2**): top leaves; (**B3**): stem base). The figures reveal the time at which the pathogen can be detected in the basal leaves, the top leaves, and the stem base after inoculation, and the optimal site for rapid detection of the pathogen detection. (**A1**–**A3**) show the results of the basal leaves, the top leaves, and the stem base, respectively. Here, green indicates a positive result, while pale orange indicates a negative result. Samples shown in (**A1**–**A3**) are as follows: 1: *Fom* (positive control); 2: deionized water (negative control); 3: DNA of healthy (non-inoculated) bitter gourd tissues (negative control); 4–10: DNA of bitter gourd tissues at 2, 4, 6, 8, 10, 12, and 15 days post-inoculation. (**B1**–**B3**) show the corresponding conventional PCR results of the basal leaves, the top leaves, and the stem base, respectively. Samples shown in (**B1**–**B3**) are as follows: M: DL1000 DNA Marker, 1: *Fom* (positive control); 2: deionized water (negative control); 3: DNA of healthy (non-inoculated) bitter gourd tissues (negative control); 4–10: DNA of bitter gourd tissues at 2, 4, 6, 8, 10, 12, and 15 days post-inoculation.

In the LAMP visualization assay, the basal leaves from 10 days after inoculation displayed a yellow-green color under natural light (Figure 9(A1)). Similarly, the top leaves from six days after inoculation (Figure 9(A2)) and the stem base from four days after inoculation (Figure 9(A3)) exhibited a yellow-green color. Meanwhile, after conventional PCR, no obvious bands were detected for the basal leaves 0–15 days after inoculation (Figure 9(B1)) or from the top leaves 0–15 days after inoculation (Figure 9(B2)). Meanwhile, faint bands were detected for the stem base eight days after inoculation, but they were not obvious. Notably, the method generated obvious bands for the stem base from 10 days after inoculation (Figure 9(B3)). These observations indicated that the established LAMP method is faster and more sensitive than the conventional PCR in detecting *Fom* in bitter gourd seedlings. Thus, the established LAMP assay could be used for the early and rapid diagnosis of bitter gourd Fusarium wilt with high specificity, sensitivity, and efficiency.

## 4. Discussion

Fusarium wilt caused by *Fusarium oxysporum* f. sp. *momordicae* (*Fom*) has become a bottleneck in bitter gourd production in China, especially in the continuous cropping greenhouses of Guangxi [6,8]. Therefore, the present study *FOC* used on developing a LAMP system to specifically detect *Fom* in bitter gourd samples, thereby supporting disease management and sustainable production.

We tested various primer sets of primers and established four core primers optimal for the method, including two outer primers (FoM-F3-1/FoM-B3-1) and two inner primers (FoM-FIP-1/FoM-BIP-1), without adding loop primers. The number of primers used in this method is consistent with that adopted in conventional LAMP technology (4–6 primers). Moreover, using Bst DNA polymerase as the core catalytic enzyme, this LAMP-based system detects the pathogen within 60 min of incubation at 64 °C. Its detection time is consistent with that of conventional LAMP, indicating no compromise in speed. The precise temperature (64 °C) used in this assay maximizes enzyme activity and accelerates the inner primer-mediated exponential amplification [15,19]. The molecular marker used in this study was derived from a *Fom*-specific genomic region located within the fragment amplified by the PCR primers FOMM-SPF/SPR. In silico analyses confirmed its low homology to other formae speciales (Table 3). Notably, this marker resides in a region adjacent to *SIX* effector genes, which are known determinants of host specificity in *F. oxysporum* [11]. Moreover, since the primer-binding sequence in LAMP was selected from the conserved regions targeted by the specific PCR primers FOMM-SPF/SPR for *Fom* and within the PCR-amplified fragments of this pathogen, non-specific binding was less. The conserved nature of the target sequence also reduced time loss by preventing non-specific amplification due to primer misbinding to non-conserved sequences. It also enabled consistent, efficient amplification across all target strains, eliminating the need for repeated optimization or retesting [15]. Thus, the assay without loop primers simplifies the workflow using an easy reagent formulation, reduces the risk of non-specific amplification [20,21], and makes it suitable for field detection [22].

Notably, the LAMP-based method produced no signal with *Fusarium oxysporum* f. sp. *niveum* (watermelon pathogen), f. sp. *melonis* (melon pathogen), f. sp. *cucumerinum* (cucumber pathogen), f. sp. *cubense* (banana pathogen), f. sp. *benincasae* (wax gourd pathogen), and *Fusarium solani*, confirming its ability to specifically recognize *Fom*. Our in silico analyses further supported this high specificity. The marker sequence showed no homologous matches in the NCBI database, and local genomic alignments with other formae speciales revealed low coverage (<45%) and numerous gaps, with no effective match in the *FOC* genome. These findings are consistent with previous studies demonstrating that different formae speciales exhibit significant structural variation and sequence polymorphism in pathogenicity-related regions, which serve as the basis for developing forma specialis-specific markers [11,12,13,14].

Furthermore, this specificity makes the assay suitable for detecting the pathogen in the field. Typically, *F. oxysporum* formae speciales exhibit strict host specificity (e.g., *F. oxysporum* f. sp. *niveum* primarily infects watermelon) and rarely cause disease in bitter gourd. However, intercropping or crop rotation systems, such as the bitter gourd-watermelon/melon/cucumerinum systems, are common in the major bitter gourd-producing regions of China (e.g., Guangxi, Guangdong, Hubei) [23,24,25]. In such fields, non-target pathogens of the intercropped plant species tend to persist in the soil of these fields as chlamydospores for multiple years [26]. This coexistence of phylogenetically related *Fusarium* taxa among non-target pathogens can potentially interfere with detection methods [27]. Moreover, the wilting symptoms caused by these non-target species are highly similar to those caused by *Fom*. Therefore, an efficient detection method should be able to distinguish the strains without misdiagnosis, thereby avoiding misdiagnosis to avoid misapplication of targeted control measures. The misapplication of fungicides and the misuse of resistant cultivars will increase production costs and fail to effectively control disease spread.

Compared with existing SCAR marker-based [28] and URP-PCR [29] methods for *Fom* detection, the LAMP assay developed in this study offers the advantages of isothermal amplification, visual detection, and field applicability, while avoiding cross-reactivity issues. In contrast, the LAMP system demonstrated breakthroughs in both specificity and practicality. The precise design of four core primers (FoM-F3-1/FoM-B3-1, FoM-FIP-1/FoM-BIP-1) helped avoid cross-reactivity with other *F. oxysporum* formae speciales. Additionally, it completes detection within 60 min under a constant temperature of 64 °C, requiring no complex instruments.

The LAMP assay was also established in this study to detect *Fom* and exhibited high sensitivity, with a detection limit of 5.6 pg/μL. This high sensitivity, combined with its demonstrated specificity, makes the assay suitable for practical field applications. To further enhance its convenience for on-site diagnosis, and to make it more convenient for field use, we incorporated a visual detection system using a fluorescent kit, allowing results to be read under both natural light and UV light without the need for specialized instruments. In this assay, the positive samples emit green fluorescence under UV light and appear green under natural light, while negative samples appear light orange. A key advantage of this visualization technique is that it requires no specialized instruments, and the results can be interpreted directly by the naked eye under natural light, enabling true point-of-care testing in field and resource-limited settings. These features align perfectly with the core principles of LAMP technology, making it an effective tool for field diagnosis [15,22].

Although real-time fluorescence LAMP provides quantitative data, it relies on expensive fluorescence detection equipment, limiting its applicability in field or basic laboratory conditions. The real-time fluorescence LAMP uses intercalating dyes (e.g., SYBR Green), which can inhibit the LAMP reaction. Moreover, probe-based methods require additional enzymes, increasing cost and reducing system stability. Given that LAMP is inherently a qualitative detection method and its core advantage is rapid, on-site diagnosis, the visual readout approach adopted in this study represents a more practical choice for field development. Moreover, this visualization technique eliminates the need for electrophoresis (conventional PCR), significantly reducing detection time, workload, and cost and improving detection efficiency. Thus, with picogram-level sensitivity and visual readout, this assay serves as a feasible tool for the early warning and precise control of bitter gourd Fusarium wilt.

In recent years, the LAMP technique has been widely used to detect pathogenic fungi, such as *Sclerotinia sclerotiorum* [30], *Aspergillus* spp. [31], *Pythium* spp. [32], *Phytophthora* spp. [33], and *Fusarium oxysporum* [16], in plant tissues without separately isolating the DNA. The 25 μL LAMP reaction system of this study detected as low as 5.6 pg/μL of the genomic DNA of *Fom*. Meanwhile, the conventional PCR assay detected only 560 pg/μL for the same target DNA, indicating higher sensitivity for the LAMP assay than the conventional PCR. Research has indicated that the sensitivity of the LAMP assay is comparable to that of the LAMP-based methods reported for detecting other *F. oxysporum* formae speciales, including *F. oxysporum* in lotus rhizomes [34], *Fusarium oxysporum* f. sp. *cubense tropical race 4* in soil [18], *Fusarium oxysporum* f. sp. *cucumerinum* in soil, plant, insects, and DNA of the known *Fusarium* species [35]. Thus, the study’s findings indicate that with a picogram-level sensitivity, LAMP can rapidly diagnose bitter gourd wilt.

In bitter gourd, wilt, caused by *Fom*, is a destructive disease with high lethality. The pathogen primarily invades via wounds on the root system or natural openings in the root epidermis and colonizes the vascular bundles, with the rhizome (root-stem transition zone) serving as the key initial accumulation site. Due to the long-distance transport and low load, the pathogen is rarely detected in the top or basal leaves during the early- to mid-stages of infection. Thus, the success of detection depends primarily on the plant part analyzed. Su et al. [36] detected the highest *F. oxysporum* concentration in the bitter gourd rhizome, followed by the root, and the lowest in the middle stem; however, no pathogen was detected in the top leaves. Therefore, the rhizome is typically used to detect the pathogen early. In this study, conventional PCR detected *F. oxysporum* f. sp. *momordicae* only in the basal stem from eight days post-inoculation (Figure 9(B3)), consistent with the findings by Su et al. [36] and Lan et al. [37]. It failed to detect the pathogen in the top or basal leaves (Figure 9(B1,B2)), which aligns with the aforementioned findings. Thus, we believe that for the early detection of *Fom*, the choice of both plant part and detection method is critical. Notably, the method used in this study enabled detection in multiple parts (top leaves, basal leaves, and basal stems) of inoculated seedlings, yielding earlier detection of the pathogen. Specifically, signals due to the pathogen were observed as early as four days post-inoculation in the basal stem (the earliest detectable part, closest to the original site of infection) and six days post-inoculation in the top leaves. Moreover, the method allowed detection of the pathogen in leaves (top/basal) via non-destructive sampling (avoiding damage to the plant’s vascular core) and in the basal stem after sampling, ensuring the earliest possible diagnosis. Thus, this study reports a LAMP assay that enables non-destructive, early detection of *Fom* in bitter gourd, with high sensitivity and efficiency. The LAMP detection system also exhibits excellent specificity and accuracy, requiring only isothermal amplification and allowing visual readout. These features provide a convenient, rapid, and non-destructive way for diagnosing a destructive plant disease. As it enables early pathogen detection, this method can be used to monitor disease occurrence and spread and support integrated disease management in the field.

## 5. Conclusions

In this study, we obtained a *Fom*-specific sequence and developed a LAMP assay based on this marker for detecting the bitter gourd wilt pathogen. The assay has four main features: (i) high specificity confirmed by both sequence analysis and cross-amplification tests; (ii) detection limit of 5.6 pg/μL, about 100 times more sensitive than conventional PCR; (iii) visual readout avoiding specialized instruments; and (iv) non-destructive early detection in asymptomatic plants. This rapid, on-site assay supports early warning, targeted scouting, and integrated disease management in intercropping/rotation systems, reducing fungicide use and yield losses. However, to enhance the assay’s applicability, future studies should validate the method with naturally infected field samples, optimize pretreatment for direct detection of the pathogen in soil/plant samples, and refine the system to improve field stability. These aspects will establish the assay as a robust tool for the sustainable control of bitter gourd wilt.

## Figures and Tables

**Table 1:** Fungal pathogens used in this study.

Number	Isolate	Scientific Name	Host	Source
1	FOM4505	*F. oxysporum momordicae*	Bitter gourd	Nanning, Guangxi, China
2	FOM4507	*F. oxysporum momordicae*	Bitter gourd	Nanning, Guangxi, China
3	FOM4514	*F. oxysporum momordicae*	Bitter gourd	Nanning, Guangxi, China
4	FOM4522	*F. oxysporum momordicae*	Bitter gourd	Wuming, Guangxi, China
5	FOM4530	*F. oxysporum momordicae*	Bitter gourd	Beihai, Guangxi, China
6	FOM4523	*F. oxysporum momordicae*	Bitter gourd	Wuming, Guangxi, China
7	FOM4552	*F. oxysporum momordicae*	Bitter gourd	Wutang, Guangxi, China
8	FOM518	*F. oxysporum momordicae*	Bitter gourd	Nanning, Guangxi, China
9	FOM4535	*F. oxysporum momordicae*	Bitter gourd	Nanning, Guangxi, China
10	FOM4582	*F. oxysporum momordicae*	Bitter gourd	Fuzhou, Fujian, China
11	FOM4598	*F. oxysporum momordicae*	Bitter gourd	Nanning, Guangxi, China
12	FOM4569	*F. oxysporum momordicae*	Bitter gourd	Nanning, Guangxi, China
13	FOM4516	*F. oxysporum momordicae*	Bitter gourd	Hengyang, Hunan, China
14	FOM4519	*F. oxysporum momordicae*	Bitter gourd	Wuming, Guangxi, China
15	FOM4527	*F. oxysporum momordicae*	Bitter gourd	Nanning, Guangxi, China
16	FOM4506	*F. oxysporum momordicae*	Bitter gourd	Nanning, Guangxi, China
17	FOM4520	*F. oxysporum momordicae*	Bitter gourd	Wuming, Guangxi, China
18	FOM4532	*F. oxysporum momordicae*	Bitter gourd	Nanning, Guangxi, China
19	FOM4578	*F. oxysporum momordicae*	Bitter gourd	Guangzhou, Guangdong, China
20	FOM4546	*F. oxysporum momordicae*	Bitter gourd	Nanning, Guangxi, China
21	FOM4577	*F. oxysporum momordicae*	Bitter gourd	Nanning, Guangxi, China
22	FOM4549	*F. oxysporum momordicae*	Bitter gourd	Nanning, Guangxi, China
23	FOM4575	*F. oxysporum momordicae*	Bitter gourd	Nanning, Guangxi, China
24	FOM4563	*F. oxysporum momordicae*	Bitter gourd	Lingchuan, Guangxi, China
25	FOM4521	*F. oxysporum momordicae*	Bitter gourd	Wuming, Guangxi, China
26	FOM4561	*F. oxysporum momordicae*	Bitter gourd	Wutang, Guangxi, China
27	FOM4536	*F. oxysporum momordicae*	Bitter gourd	Nanning, Guangxi, China
28	FOM4525	*F. oxysporum momordicae*	Bitter gourd	Nanning, Guangxi, China
29	FOM4550	*F. oxysporum momordicae*	Bitter gourd	Wutang, Guangxi, China
30	FOM4558	*F. oxysporum momordicae*	Bitter gourd	Wutang, Guangxi, China
31	FOM4553	*F. oxysporum momordicae*	Bitter gourd	Wutang, Guangxi, China
32	FOM4528	*F. oxysporum momordicae*	Bitter gourd	Nanning, Guangxi, China
33	FOM4567	*F. oxysporum momordicae*	Bitter gourd	Nanning, Guangxi, China
34	FOM4571	*F. oxysporum momordicae*	Bitter gourd	Nanning, Guangxi, China
35	FOM4509	*F. oxysporum momordicae*	Bitter gourd	Nanning, Guangxi, China
36	FOM4572	*F. oxysporum momordicae*	Bitter gourd	Nanning, Guangxi, China
37	FOM4501	*F. oxysporum momordicae*	Bitter gourd	Nanning, Guangxi, China
38	FOM4503	*F. oxysporum momordicae*	Bitter gourd	Nanning, Guangxi, China
39	FOM4513	*F. oxysporum momordicae*	Bitter gourd	Nanning, Guangxi, China
40	FOM4529	*F. oxysporum momordicae*	Bitter gourd	Nanning, Guangxi, China
41	FOM4517	*F. oxysporum momordicae*	Bitter gourd	Nanning, Guangxi, China
42	FOM4534	*F. oxysporum momordicae*	Bitter gourd	Nanning, Guangxi, China
43	FOM4515	*F. oxysporum momordicae*	Bitter gourd	Nanning, Guangxi, China
44	FOM4533	*F. oxysporum momordicae*	Bitter gourd	Yulin, Guangxi, China
45	ACCC30024	*F. oxysporum niveum*	Watermelon	ACCC
46	Xi1	*F. oxysporum niveum*	Watermelon	Nanning, Guangxi, China
47	Xi2	*F. oxysporum niveum*	Watermelon	Beihai, Guangxi, China
48	Xi3	*F. oxysporum niveum*	Watermelon	Guangzhou, Guangdong, China
49	Tian1	*F.oxysporum melonis*	Melon	Laibing, Guangxi, China
50	ACCC30220	*F. oxysporum cucumerinum*	Cucumber	ACCC
51	Saigon banana FOP1	*F. oxysporum cubense*	Saigon banana	Nanning, Guangxi, China
52	Dongku-2	*F.oxyspoorum benincasae*	Wax gourd	Nanning, Guangxi, China
53	Nandongku 106	*F.oxyspoorum benincasae*	Wax gourd	Nanxiao, Guangxi, China
54	CGMCC3.4.604	*F. solani*	Eggplant	CGMCC

**Table 2:** Sequences of the primers used for LAMP and PCR in this study.

Group	Primers	Sequence (5′–3′)
FoM-1-1	FoM-F3-1	CGAGGCTAGCTAGCGTGA
	FoM-B3-1	CTCTAGAGGCGAGGGAGAG
	FoM-FIP-1	AGCTGATGGCTCGACGAGCTGAGGATGCTCTTTGCCAACC
	FoM-BIP-1	CCTGTTTTTGTAGCACCACCGCTGCTCGGCAGAGAACATCT
	FoM-LB-1	AGATTGTTGAGGCTCATAAGCGT
FoM-1-2	FoM-F3-1	CGAGGCTAGCTAGCGTGA
	FoM-B3-1	CTCTAGAGGCGAGGGAGAG
	FoM-FIP-1	AGCTGATGGCTCGACGAGCTGAGGATGCTCTTTGCCAACC
	FoM-BIP-1	CCTGTTTTTGTAGCACCACCGCTGCTCGGCAGAGAACATCT
FoM-3-1	FoM-F3-3	TCAGCTCTGCCTCTTCGT
	FoM-B3-3	GCGTCAACAATCTTGCGATAG
	FoM-FIP-3	CGGCAGAGAACATCTAGACACGCCCCCTGTTTTTGTAGCACCA
	FoM-BIP-3	ACCCTCTCCCTCGCCTCTAGGCTACAGAACCAGGGGAATT
	FoM-LF-3	TGAGCCTCAACAATCTTGCGG
	FoM-LB-3	CTCGTCGAGCCATCTGCTC
FoM-3-2	FoM-F3-3	TCAGCTCTGCCTCTTCGT
	FoM-B3-3	GCGTCAACAATCTTGCGATAG
	FoM-FIP-3	CGGCAGAGAACATCTAGACACGCCCCCTGTTTTTGTAGCACCA
	FoM-BIP-3	ACCCTCTCCCTCGCCTCTAGGCTACAGAACCAGGGGAATT
	FoM-LF-3	TGAGCCTCAACAATCTTGCGG
FoM-3-3	FoM-F3-3	TCAGCTCTGCCTCTTCGT
	FoM-B3-3	GCGTCAACAATCTTGCGATAG
	FoM-FIP-3	CGGCAGAGAACATCTAGACACGCCCCCTGTTTTTGTAGCACCA
	FoM-BIP-3	ACCCTCTCCCTCGCCTCTAGGCTACAGAACCAGGGGAATT
PCR	FOMM—SPF	AAGGATAACGAGGCTAGCT
	FOMM—SPR	GTATAGAGCATCTAGACACGAATGC

**Table 3:** Sequence characteristics and in silico specificity validation of the *Fom*-specific marker.

Feature	Description
Marker sequence (5′→3′)	AAGGATAACGAGGCTAGCTAGCGTGAGAGGATGCTCTTTGCCAACCACCCTTTCATTCCCCTCAAGAGCTCGTCGAGCCATCAGCTCTGCCTCTTCGTGTTTCCCCTGTTTTTGTAGCACCACCGCAAGATTGTTGAGGCTCATAAGCGTGTCTAGATGTTCTCTGCCGAGCACCCTCTCCCTCGCCTCTAGAGCTCGTCGAGCCATCTGCTCGGCTTCCTCAAAATTCCCCTGGTTCTGTAGCACTATCGCAAGATTGTTGACGCTTGCATTCGTGTCTAGATGCTCTATAC
Length	293 bp
NCBI GenBank BLAST (E-value ≤ 1 × 10^−5^, coverage ≥80%)	No significant homologous matches found
Local alignment with other *F. oxysporum* formae speciales (BLASTn, E-value ≤ 1 × 10^−5^)	Coverage consistently <45% with multiple gaps No valid match detected in one forma specialis (similarity <60%)

Note: Local alignments were performed against genome sequences of standard strains obtained from the Agricultural Culture Collection of China. The pronounced sequence differences confirm the marker’s specificity at the genomic level, consistent with previous studies demonstrating significant structural variation in pathogenicity-related regions among different formae speciales [11,12,13,14].

## Data Availability

The original contributions presented in this study are included in the article. Further inquiries can be directed to the corresponding authors.
